# A Randomized, Double‐Blind, Two‐Treatment, Two‐Period, Crossover Study Investigating the Systemic Bioavailability of a Novel Cocrystal Ubiquinol Formulation Compared with a Ubiquinone Formulation in Healthy Adults

**DOI:** 10.1002/cpdd.70042

**Published:** 2026-03-06

**Authors:** Xuefeng Mei, Bingqing Zhu, Kshitij Soni, Kishore Kasaraneni, Nirav Panchal

**Affiliations:** ^1^ Shanghai Institute of Materia Medica Chinese Academy of Sciences Shanghai China; ^2^ Cocrystal Health Industry Co., Ltd Zhejiang China; ^3^ Credevo Pte. Ltd. Singapore Singapore

**Keywords:** bioavailability, coenzyme Q10, CoQ10 supplementation, pharmacokinetics, ubiquinol cocrystal, ubiquinone

## Abstract

Coenzyme Q10 (CoQ10) is a naturally occurring biochemical cofactor found in all human cell membranes in two interconvertible forms: oxidized ubiquinone and reduced ubiquinol. Clinical studies indicate that different CoQ10 formulations have different absorption rates, highlighting research comparing their systemic bioavailability. This study compared the oral bioavailability of cocrystal formulation soft gels (test product), a novel ubiquinol formulation, and ubiquinone formulation (reference product) in a randomized, double‐blind, two‐period crossover study with 12 healthy subjects under fasting conditions. The secondary objective of this study was to evaluate the safety and tolerability of the ubiquinol formulation. The pharmacokinetic analyses indicated that the test ubiquinol formulation demonstrated substantially higher relative systemic bioavailability compared with the ubiquinone reference. The geometric mean ratios (test/reference) for baseline‐corrected peak plasma concentration (C_max_) and area under the curve from zero to last quantifiable time (AUC_0–t_) were 2.20 and 2.01, respectively, with 90% confidence intervals of 1.59–3.04 and 1.51–2.70. The geometric mean ratio for AUC from time zero to infinity (AUC_0–∞_) was 3.43 (90% CI: 1.47–8.00). No adverse events were reported in this small pilot study for either of the formulations. These findings demonstrate that ubiquinol has a better systemic bioavailability than ubiquinone, supporting the novel formulation's potential as a promising alternative to traditional CoQ10 supplements.

Coenzyme Q10 (CoQ10), commonly known as ubiquinone, is a fat‐soluble, vitamin‐like substance found in human cell membranes and other aerobic organisms such as bacteria and mammals.^1^ CoQ10 transfers electrons from complexes I and II to complex III of the mitochondrial respiratory chain, cycling between reduced (ubiquinol) and oxidized (ubiquinone) forms.^2^ CoQ10 is the body's only lipophilic antioxidant and is necessary for mitochondrial bioenergetics.^3^ Its redox characteristics neutralize reactive oxygen species, preventing oxidative stress, DNA and protein degradation, and oxidative lipid damage.^4^ It is especially common in organs such as the liver, kidneys, heart, and brain, which require a lot of energy.

CoQ10 has been associated with several potential health benefits, including enhanced endothelial function, a reduction in significant adverse cardiovascular events, and improved cardiac health in individuals with heart failure.^5^, ^6^, ^7^, ^8^, ^9^ It may also help with muscle pain caused by statin drugs, as well as migraines, mitochondrial disorders, fibromyalgia, and type 2 diabetes.^10^, ^11^, ^12^, ^13^ CoQ10 has also been shown to improve general metabolic and reproductive health in individuals with polycystic ovarian syndrome, improve erectile function, insulin sensitivity, and hormonal balance.^14^, ^15^

Research suggests that the redox conversion from ubiquinone to ubiquinol becomes less efficient in elderly populations. This lowers CoQ10 levels and weakens the body's antioxidant defense.^16^, ^17^ Adding ubiquinol to the diet has been shown to raise plasma CoQ10 levels more quickly and to a greater extent than adding ubiquinone, especially in older adults.^16^ Consequently, ubiquinol supplementation may provide improved antioxidant protection and support mitochondrial function in elderly individuals and those with impaired health.

Studies indicate that the administration of ubiquinol significantly increases plasma CoQ10 levels in healthy subjects compared to an equivalent dosage of ubiquinone.^18^ In individuals with advanced congestive heart failure, ubiquinol supplementation markedly elevated plasma CoQ10 levels and correlated with enhanced cardiac performance.^19^

The principal challenge with ubiquinol, however, is its oxidative instability, which leads to rapid conversion to ubiquinone under ambient conditions. This chemical reactivity complicates long‐term storage and limits its incorporation into various pharmaceutical dosage forms.^20^, ^21^ A recent study by Zhang et al reported a cocrystal of ubiquinol with nicotinamide. This cocrystal demonstrated excellent stability, improved dissolution properties, and higher bioavailability compared to conventional ubiquinol.^22^ The cocrystal remained stable for an extended period, even when stored under stressed conditions (40°C, 75% relative humidity). A pharmacokinetic study in rats further showed that the cocrystal had a 4.5 times greater AUC_0–t_ than conventional ubiquinol.

Cocrystal formulation soft gels have been developed using cocrystal ubiquinol, offering a stable ubiquinol formulation with enhanced bioavailability. This study evaluates the oral bioavailability of cocrystal formulation soft gels (test product) compared to ubiquinone formulation (reference product), aiming to confirm its potential as a promising CoQ10 supplement in humans.

## Methods

### Ethical Approval

This study was executed in accordance with the International Council for Harmonisation Good Clinical Practice (ICH‐GCP) principles, the latest edition of the Declaration of Helsinki, and relevant regulatory mandates, including the archiving of essential records. The final study protocol, informed consent forms in English and Gujarati, screening case report forms, study case report forms, and all related study documents were reviewed and approved by the Conscience Independent Ethics Committee (CIEC), Ahmedabad, Gujarat, India. Informed written consent was acquired from all study participants before screening and enrollment.

### Selection of Study Participants

Twelve healthy adults (6 males and 6 females), aged 45–65 years with a BMI of 18.5–30.0 kg/m^2^ and body weight ≥48 kg, were enrolled in the study. Participants were classified as healthy based on their baseline medical history, vital signs, physical examination, chest x‐ray, 12‐lead electrocardiography (ECG), hematological analysis, biochemical assessment, lipid profile, and urinalysis; female participants also underwent urine pregnancy tests.

The inclusion criteria consisted of healthy human subjects, a non‐smoker status, no prior history of alcoholism or substance abuse, and the participant's commitment to abstaining from alcohol, tobacco, and xanthine‐containing foods, both before and throughout the duration of the study. Subjects were excluded if they had a known hypersensitivity to ubiquinol or formulation excipients; required medications with enzyme‐modifying activity within 28 days; or had taken prescription drugs, over‐the‐counter products, or supplements (including vitamins and minerals) within 14 days before dosing. Individuals with a history of cardiovascular, hepatic, renal, gastrointestinal, neurological, psychiatric, or other clinically significant diseases; malignancy; swallowing difficulties; or any condition interfering with gastrointestinal or hematopoietic function were not eligible.

### Study Design

This was a randomized, double‐blind, balanced, two‐treatment, two‐period, two‐sequence, single‐dose crossover bioavailability study conducted in healthy adult volunteers. The two treatments were a single oral dose of the test formulation (T; cocrystal formulation 120 mg soft gels, 2 × 120 mg) and the reference formulation (R; ubiquinone formulation 120 mg soft gels, 2 × 120 mg). The test product (T) contains a cocrystal of ubiquinol as the active pharmaceutical ingredient while the reference product (R) contains standard ubiquinone. The order of receiving the investigational product for each subject was determined according to a randomization schedule. Subjects were randomized to one of the two sequences: either TR or RT. Equal allocation of the sequence was ensured. The randomization schedule was generated by using PROC PLAN SEED procedure in SAS software 9.4 version. A single oral dose of T or R was administered with 240 ± 2 mL of water at room temperature under fasting conditions. Subjects underwent fasting for a minimum of 10 h before dosing and for at least 4 h following the dose in each study period. Blood collection was performed in a series for up to 48 h to assess the pharmacokinetic profile. A 14‐day washout period was considered between periods to prevent carryover effects, given the elimination half‐life of ubiquinol (approximately 30 h).

### Plasma Drug Concentration Analysis

Blood samples were taken from participants to evaluate the pharmacokinetics (PK) of the study drug. A total of 12 samples were collected from each subject during each study period. The post‐dose samples measured 3 mL, while the pre‐dose samples measured 5 mL. Sampling was conducted at three intervals before dosing: −1.0, −0.5, and 0 h (collected within 5 min before administration). Additionally, post‐dose samples were taken at 1, 2, 4, 6, 8, 10, 12, 24, and 48 h. The time of blood collection was noted for the calculation of the PK parameters. K_2_EDTA‐coated vacutainer tubes were used for the collection of blood samples and were maintained in a wet ice bath at ≤4°C before processing. To separate the plasma, samples were centrifuged at 3500 rpm at 4°C ± 2°C for 5 min. The separated plasma was transferred into a pre‐labeled amber polypropylene tube and split into two parts: one for analysis (∼0.4 mL, or 0.7 mL for pre‐dose samples) and the other for control, with the rest of the volume. The samples were stored in a deep freezer at −20°C ± 5°C or lower. Sample collection, processing, separation, and storage were performed under sodium vapor light conditions.

To quantify the total ubiquinone plasma concentrations, a validated liquid chromatography‐tandem mass spectrometry (LC‐MS/MS) technique was used. Sample preparation was done at room temperature under yellow monochromatic light. The 100 µL aliquots of plasma were placed in labeled tubes. Each sample was added with 50 µL of an internal standard solution (ubiquinone‐d6 in 1‐propanol, 1000 ng/mL), except for the standard blank, blank QC, and samples that used 50 µL of 1‐propanol instead, followed by vortexing for a minimum of 30 s. To ensure the complete conversion of ubiquinol to ubiquinone, 100 µL of p‐benzoquinone solution (1 mg/mL in 1‐propanol) was then added, vortexed, and left at room temperature for 15 min. Following incubation, 500 µL of 1‐propanol was added to the samples and vortexed for 5 min. The tubes were centrifuged at 14,000 rpm for 5 min at 10°C ± 2°C. The supernatant was then transferred to autosampler vials for analysis.

Separation was carried out using a Zorbax SB‐AQ column (150 × 4.6 mm, 5 µm), at 30°C. The mobile phase consisted of (10 mM ammonium formate in [methanol:1‐propanol; 90:10v/v]: 0.1% formic acid solution in water [v/v]:99:1v/v) and was delivered at a flow rate of 0.8 mL/min under isocratic conditions. The autosampler was maintained at 6°C, and the injection volume was 5 µL.

The analysis was performed using a SCIEX 4500 triple quadrupole mass spectrometer equipped with an electrospray ionization (ESI) source in positive ion mode. The detection was carried out in multiple reaction monitoring (MRM) mode, monitoring the transitions for ubiquinone (m/z 863.60 → 197.10) and the internal standard (ubiquinone‐d6) (m/z 886.90 → 203.10). The quantification was determined by the ratio of the analyte's peak area to that of the internal standard.

The calibration curve was linear over the validated concentration range of 30.0 to 12,000.0 ng/mL using a 1/x^2^ weighting. The lower limit of quantification (LLOQ) was 30.0 ng/mL. Quality control (QC) samples were prepared at low, medium, and high concentrations within this range. For intra‐ and inter‐batch accuracy and precision, the acceptance limits were within the ranges (±15% for QC levels, ±20% at LLOQ). The method was confirmed to be free of significant carryover, cross‐contamination, and matrix effects. The stability of the analyte was confirmed under both storage and processing conditions.

### Safety Evaluation

All adverse events (AEs) were monitored throughout the study. Safety assessments included physical examination, vital signs (blood pressure, pulse rate, and body temperature), 12‐lead ECGs, and laboratory investigations (hematology, serum chemistry, lipid profile, and urinalysis). Well‐being assessments and vital signs were measured at scheduled time points pre‐dose (within 3 h before dosing) and post‐dose. Safety evaluations were repeated at discharge and during follow‐up, including physical examination, vital signs, lipid profile tests, hemogram, and biochemistry tests.

### Pharmacokinetic Analysis

Pharmacokinetic assessments were carried out using data from all participants who completed the study. Statistical evaluations were performed using a noncompartmental analysis approach with SAS software (version 9.4). The primary PK measures were the peak plasma concentration (C_max_), the area under the plasma concentration–time curve up to the last quantifiable sample (AUC_0−t_), area under the plasma concentration–time curve from time zero to infinity (AUC_0–∞_), time to reach maximum concentration (T_max_), and terminal elimination half‐life (t_1/2_). Observed blood sampling times were used for all analyses, and no imputation was applied for missing plasma concentrations. The relative bioavailability (F_T/R_) of the test formulation compared with the reference was estimated using geometric mean ratios (Test/Reference) with corresponding 90% confidence intervals (CIs) for ln‐transformed C_max_, AUC_0–t_, and AUC_0–∞_ values derived from mixed‐effects models consistent with standard PK study reporting. The sample size of 12 subjects (6 per sequence) was selected for this pilot study, reflecting a design to explore preliminary bioavailability differences based on in vivo studies data suggesting a significant increase.^22^ Given the high variability in CoQ10 absorption, this sample size supports preliminary findings to support larger trials.

For baseline correction, the mean of the three pre‐dose concentrations (−1.0, −0.5, and 0 h) was calculated for each subject in each period. This mean baseline value was then subtracted from all post‐dose plasma concentrations, following standard practice for endogenous compounds.

AUC_0–t_ was determined using the linear trapezoidal method up to the last quantifiable concentration (C_last_). AUC_0–∞_ was calculated as AUC_0–t_ + C_last/λz_, where λz is the terminal elimination rate constant derived from the log‐linear portion of the concentration–time curve.

## Results

### Study Participants

Twelve healthy adult volunteers were enrolled in the study, and all subjects completed both study periods under fasting conditions. No subjects discontinued after randomization; therefore, 12 participants were included in the PK and statistical analyses.

### Pharmacokinetics

Baseline‐corrected and baseline‐uncorrected plasma concentrations of total ubiquinone were analyzed. Key pharmacokinetic parameters (arithmetic mean ± SD, median, and range) are summarized in Table 1 (baseline corrected) and Table 2 (baseline uncorrected).

**Table 1:** Summary of Baseline‐Corrected Pharmacokinetic Parameters of Ubiquinol (Test Product, T) and Ubiquinone (Reference Product, R)

**Parameter**	**Test Product (T)** **Mean ± S.D,** **Median (Range)**	**Reference Product (R)** **Mean ± S.D,** **Median (Range)**
C_max_ (µg/mL)	1.041 ± 0.585 Median: 1.005 (0.319–2.134)	0.486 ± 0.314 Median: 0.407 (0.138–1.139)
T_max_ (h)	Median: 6.00 (6.0–8.0)	Median: 6.00 (6.0–48.0)
AUC_0–t_ (h µg/mL)	20.249 ± 10.724 Median: 19.679 (5.143–44.402)	10.253 ± 5.616 Median: 9.498 (2.447–17.605)
AUC_0–inf_ (h µg/mL)	38.636 ± 28.892 Median: 33.222 (8.149–106.051)	17.246 ± 10.016 Median: 20.371 (3.050–28.020)**
t½ (h)	36.8 ± 23.4 Median: 33.0 (13.3–96.5)	30.2 ± 16.7 Median: 36.3 (6.8–52.6)**
Residual area (%)	0.4 ± 0.2 Median: 0.4 (0.08–0.73)	0.3 ± 0.2 Median: 0.4 (0.01–0.6)**
AUC_extrap_ (%)	38.8 ± 17.6 Median: 40.0 (8.4–72.8)	31.3 ± 20.7 Median: 37.8 (1.2–59.6)**
AUC_0–t_/AUC_0–inf_	0.6 ± 0.2 Median: 0.6 (0.3–0.9)	0.7 ± 0.2 Median: 0.6 (0.4–1.0)**

AUC_0–inf_, area under the plasma concentration–time curve from time zero extrapolated to infinity; AUC_0–t_, area under the plasma concentration–time curve from time zero to the last measurable concentration; AUC_0–t_/AUC_0–inf_, ratio of observed to extrapolated AUC; AUC_extrap_, percentage of AUC extrapolated beyond the last measurable concentration; C_max_, maximum observed plasma concentration; t_½_, terminal elimination half‐life; Residual area, proportion of the extrapolated area relative to total AUC; T_max_, time to reach C_max_.

*Note*: No value of AUC_0–inf_, AUC_0–t_/AUC_0–inf_, or t_1/2_ will be reported for cases that do not exhibit a terminal log‐linear phase in the concentration versus time profile.

** Reference n = 7 subjects.

**Table 2:** Summary of Baseline‐Uncorrected Pharmacokinetic Parameters of Ubiquinol (Test Product, T) and Ubiquinone (Reference Product, R)

Parameter	Test Product (T)	Reference Product (R)
C_max_ (µg/mL)	1.784 ± 0.580 Median: 1.704 (0.767–2.719)	1.210 ± 0.537 Median: 1.005 (0.562–2.167)
T_max_ (h)	Median: 6.00 (6.00–8.00)	Median: 6.00 (6.0–48.0)
AUC_0–t_ (h µg/mL)	56.014 ± 16.652 Median: 55.325 (28.640–98.505)	44.936 ± 16.953 Median: 39.277 (23.299–76.136)
AUC_0–inf_ (h µg/mL)	221.183 ± 121.032 Median: 187.025 (83.998–463.968)	956.974 ± 2059.046 Median: 195.849 (83.195–6043.880)**
t½ (h)	106.9 ± 50.3 Median: 98.1 (48.4–224.8)	633.2 ± 1354.0 Median: 165.0 (96.1–3982.3)**
Residual area (%)	0.7 ± 0.1 Median: 0.7 (0.5–0.9)	0.8 ± 0.1 Median: 0.8 (0.7–1.0)**
AUC_extrap_ (%)	70.5 ± 10.2 Median: 72.0 (50.9–86.7)	81.9 ± 9.1 Median: 82.1 (70.2–99.2)**
AUC_0–t_/AUC_0–inf_	0.30 ± 0.10 Median: 0.3 (0.1–0.5)	0.2 ± 0.1 Median: 0.2 (0.01–0.3)**

AUC_0–inf_, area under the plasma concentration–time curve from time zero extrapolated to infinity; AUC_0–t_, area under the plasma concentration–time curve from time zero to the last measurable concentration; AUC_0–t_/AUC_0–inf_, ratio of observed to extrapolated AUC; AUC_extrap_, percentage of AUC extrapolated beyond the last measurable concentration; C_max_, maximum observed plasma concentration; T_max_, time to reach C_max_; Residual area, proportion of the extrapolated area relative to total AUC; t_½_, terminal elimination half‐life.

*Note*: No value of AUC_0–inf_, AUC_0–t_/AUC_0–inf_, or t_1/2_ will be reported for cases that do not exhibit a terminal log‐linear phase in the concentration versus time profile.

** Reference n = 8.

### Baseline‐Corrected Data

The test product demonstrated substantially higher plasma exposure to total ubiquinone compared with the reference. For baseline corrected, the geometric least‐squares mean (LSM) ratio (Test/Reference) for C_max_ was 2.20 (90% CI: 1.59–3.04), for AUC_0–t_ was 2.01 (90% CI: 1.51–2.70), and for AUC_0–∞_ was 3.43 (90% CI: 1.47–8.00). Intra‐subject variability (coefficient of variation [CV%]) was 46.2% for C_max_, 40.9% for AUC_0–t_, and 61.8% for AUC_0–∞_, with post hoc power analyses indicating 29.1% for C_max_, 34.1% for AUC_0–t_, and 10.2% for AUC_0–∞_, reflecting the study's limited power due to high variability in this dataset (Table 3).

**Table 3:** Statistical Comparison of Pharmacokinetic Parameters of Ubiquinol (Test Product, T) and Ubiquinone (Reference Product, R) for Both Baseline Corrected and Baseline Uncorrected. Results Are Presented as Geometric Least‐Squares Means (LSM), Geometric LSM Ratios (Test/Reference, %), 90% Confidence Intervals (CI), and Intra‐Subject Coefficient of Variation (CV%)

Baseline Value	Pharmacokinetic Parameter	Geometric LSM Test (T)	N_T_	Geometric LSM Reference (R)	N_R_	Geometric LSM Ratio (%)	90% CI	Intra‐Subject CV%	Power (%)
Baseline corrected	C_max_ (µg/mL)	0.886	12	0.402	12	220.2	159.1–304.9	46.2	29.1
	AUC_0–t_ (h µg/mL)	17.328	12	8.582	12	201.9	151.0–270.0	40.9	34.1
	AUC_0–inf_ (h µg/mL)	29.612		8.613		343.8	147.6–800.6	61.8	10.2
Baseline uncorrected	C_max_ (µg/mL)	1.692	12	1.106	12	153.0	124.5–188.0	28.4	55.8
	AUC_0–t_ (h µg/mL)	53.870	12	42.047	12	128.1	115.0–142.8	14.7	95.8
	AUC_0–inf_ (h µg/mL)	193.597	12	438.809	12	44.1	22.9–85.1	71.7	12.9

CI, confidence interval; CV, coefficient of variation; LSM, least‐squares mean; NT, NR; number of subjects included in 90% CI calculation for test and reference formulations, respectively.

The individual baseline‐corrected C_max_ values and AUC_0–t_ are presented in a spaghetti plot (Figure S1), showing the variability and direction of change between the Test and Reference formulations for each subject across both treatment sequences. Table S1 provides the pharmacokinetic parameters adjusted by individual baselines for each subject. One individual (subject 4) showed a significant increase in exposure, with baseline‐corrected AUC_0–t_ values approximately eight times higher than the lowest observed exposure, thereby illustrating increased systemic bioavailability in that individual.

**Figure 1:**
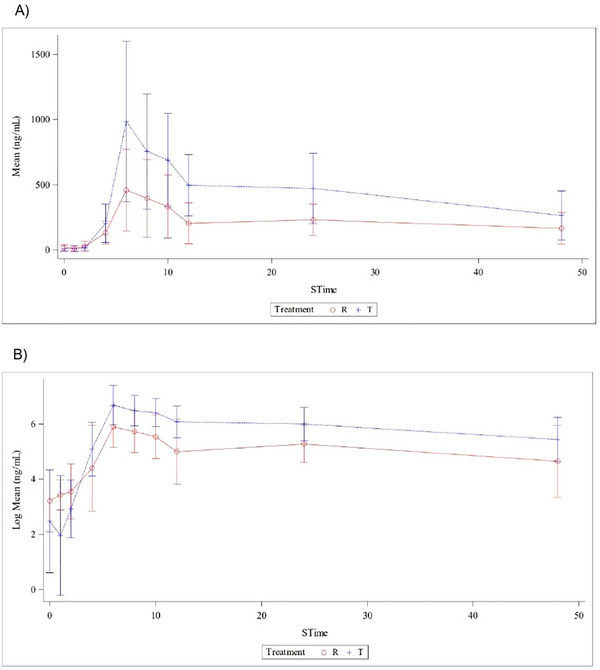
Overview of mean plasma concentration–time profiles of baseline‐corrected total ubiquinone following administration of the test (T, blue) and reference (R, red) formulations following single‐dose administration in healthy subjects. (A) Linear mean plasma concentration–time curves (mean ± SD, ng/mL). (B) Log‐transformed mean plasma concentration–time curves (log mean ± SD, ng/mL). Error bars represent the standard error of the mean. The plots illustrate comparative absorption and elimination trends between the two formulations across the sampling period.

Mean plasma concentration–time profiles indicated higher peak and overall exposure for the test product across the sampling interval. Semi‐logarithmic plots confirmed prolonged plasma exposure, while linear plots clearly demonstrated peak concentrations and elimination phases (Figure 1A,B). Treatment effects were significant for both C_max_, AUC_0–t_, and AUC_0–∞_, whereas no significant period effect and only a minimal sequence effect were observed for C_max_.

The minimal sequence effect was statistically negligible, suggesting no carryover or insufficient washout with the 14‐day interval, which exceeds the elimination half‐life of ubiquinol (∼30 h).

### Baseline‐Uncorrected Data

In the uncorrected analysis, the Test/Reference geometric LSM ratio was 1.53 (90% CI: 1.24–1.88) for C_max,_ 1.28 (90% CI: 1.15–1.42) for AUC_0–t,_ and 0.44 (90% CI: 0.22‐0.85) for AUC_0–∞_. The intra‐subject variability for C_max_ was 28.4%, for AUC_0–t_ it was 14.7%, and for AUC_0–∞_ it was 71.7% with post hoc power analyses showing 55.8% for C_max_, 95.8% for AUC_0–t_, and 12.9% for AUC_0–∞_ (Table 3). The pharmacokinetic parameters of each subject's individual baseline without correction are summarized in Table S2, providing a comparison with the baseline‐corrected analysis.

**Figure 2:**
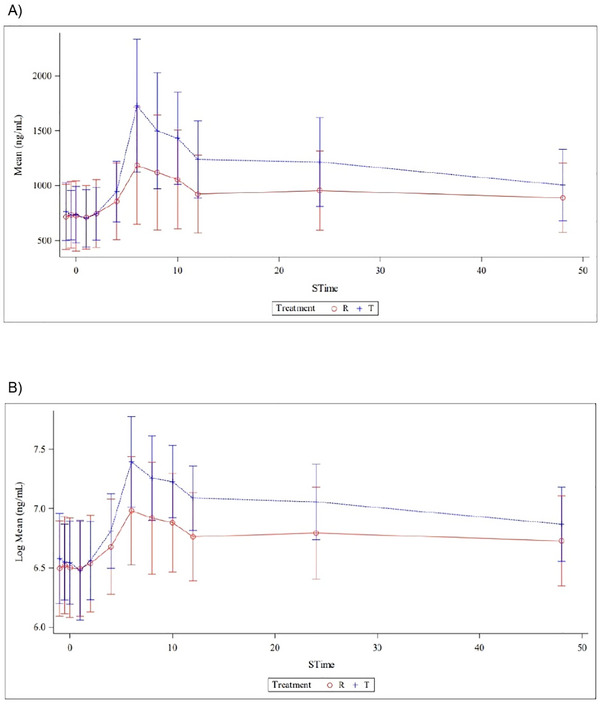
Overview of mean plasma concentration–time profiles of baseline‐uncorrected total ubiquinone following administration of the test (T, blue) and reference (R, red) formulations following single‐dose administration in healthy subjects. (A) Linear mean plasma concentration–time curves (mean ± SD, ng/mL). (B) Log‐transformed mean plasma concentration–time curves (log mean ± SD, ng/mL). Error bars represent the standard error of the mean. The plots illustrate comparative absorption and elimination trends between the two formulations across the sampling period.

The plasma concentration–time profiles demonstrated consistent trends, with the test product exhibiting higher plasma concentrations than the reference in all instances (Figure 2A,B). The treatment effects were statistically significant for both C_max_ and AUC_0–t_, and AUC_0–∞_, and no notable sequence or period effects were observed.

The absence of sequence or period effects supports the robustness of the study design and indicates that the observed differences are inflicted by formulation performance.

### Safety and Tolerability

No adverse events, serious or significant adverse events, were reported during the entire course of the study. No clinically meaningful changes were noted in laboratory parameters, ECGs, or vital signs. These findings confirm the safety and tolerability of both ubiquinol and ubiquinone formulations under the study conditions.

## Discussion

This study compared the PK and safety profiles of a test cocrystal ubiquinol formulation with a ubiquinone formulation after oral administration under fasting conditions. After correcting baseline data, ubiquinol was found to be about 2.2 times the C_max_ and 2.0 times the AUC_0–t_ of ubiquinone. AUC_0–∞_ further confirmed enhanced bioavailability with a 3.4‐fold increase, representing total systemic exposure. This showed that plasma concentration levels of the test ubiquinol were significantly higher than those of ubiquinone in the study participants.

CoQ10 is a lipophilic molecule essential to mitochondrial energy production, especially for organs with high metabolic needs such as the heart, kidneys, and liver. Research shows that advanced liposomal and water‐soluble formulations significantly increase bioavailability and clinical effectiveness, despite oral consumption often being hampered by low water solubility.^23^ For example, in the context of neurodegenerative disorders, the limited oral bioavailability, the poor solubility, and the limited transfer over the blood–brain barrier are important factors contributing to moderate or inconsistent efficacy. Formulations, such as ubiquinol, have a significant clinical advantage in boosting absorption and achieving better plasma and tissue concentrations at lower doses and shorter treatment intervals.^18^ This is especially important in chronic diseases that require prolonged supplementation. For ubiquinone to work in the body, it needs to be converted into ubiquinol, the active form of the antioxidant, in the blood or intestines. This conversion process can differ considerably, particularly among older adults and individuals with specific chronic conditions.^23^ This phenomenon may be linked to the varying efficiency of the enzymatic system responsible for converting ubiquinone to ubiquinol. For individuals with a weaker conversion capacity, direct supplementation with ubiquinol can bypass this inefficient step, leading to a substantial increase in bioavailability. Clinical studies suggest that the circulation of ubiquinol levels is associated with improved endothelial function, reduced oxidative stress, and symptoms of cardiovascular disease.^8^ They also help in reducing muscle symptoms in patients receiving statin therapy. The benefits depend on having sufficient levels of ubiquinol in tissues, which can be difficult to achieve with poorly absorbed formulations. Therefore, improved absorption directly leads to better efficacy and consistent clinical results. Thus, using ubiquinol as a form of CoQ10 supplement not only generally improves absorption efficiency but also offers more significant health benefits to a specific population.

Therefore, using ubiquinol as a form of CoQ10 supplementation not only generally improves absorption efficiency but also provides more significant health benefits for specific populations.

In this study, approximately 60% of participants demonstrated a significant increase in bioavailability with ubiquinol, with exposure increments ranging from 2.4 to 8 times. This variability corresponds with prior studies demonstrating that endogenous reductase activity and formulation factors can affect inter‐individual variations in CoQ10 absorption.^24^ This study shows that ubiquinol achieves higher absorption than ubiquinone, indicating its potential for greater clinical benefits. Prior clinical studies have examined CoQ10 supplementation across diverse disease categories, including cardiovascular diseases, neurodegenerative disorders, and mitochondrial diseases. A study involving patients with moderate dyslipidemia indicated that CoQ10 supplementation enhanced endothelial function, thereby corroborating its cardiovascular advantages.^25^, ^26^ Studies suggest that plasma CoQ10 levels above 2.5–3.0 µg/mL are associated with measurable improvements in endothelial function and oxidative stress markers.^19^ However, conventional ubiquinone supplements often fail to achieve these threshold concentrations in a substantial proportion of populations, particularly in elderly individuals and those with impaired gastrointestinal absorption or reduced enzymatic conversion capacity. As reviewed by Cirilli and colleagues, from a clinical perspective, a formulation with higher bioavailability is relevant because the proposed therapeutic effects of CoQ10 depend on achieving sufficient and reproducible systemic and tissue concentrations.^27^ The beneficial actions attributed to CoQ10, including improvement of endothelial function through enhanced nitric oxide bioavailability and reduction of oxidative stress and inflammation, are concentration dependent. Endothelial dysfunction in cardiovascular disease is characterized by a decrease in the availability of nitrogen oxides and an increase in reactive oxygen species. However, insufficient delivery of vascular tissues may limit these effects.

Likewise, the low bioavailability of CoQ10 remains a major limitation in terms of diabetes and its complications, especially diabetic neuropathy.^27^ Greater bioavailability can lead to improved antioxidant capacities and mitochondrial support in metabolically stressed tissues. The clinical importance of improved bioavailability is further highlighted by several promising treatments for CoQ10.

Preliminary studies suggest the benefits of adding CoQ10 after cardiac surgery in patients with chronic heart failure and mitochondrial cytopathy.^28^, ^29^, ^30^, ^31^, ^32^ For these patient populations to benefit from CoQ10 supplementation, formulations must reliably deliver the compound to target tissues at concentrations sufficient to exert therapeutic effects.

In summary, although CoQ10 is classified as a supplement, it is clinically and pharmaceutically critical to achieve higher bioavailability. It is widely used for therapeutic purposes, including cardiovascular health, mitochondrial support, and metabolic conditions, so it is necessary to design formulations that can reliably provide clinically significant plasma and tissue concentrations.

In a separate systematic review of type 2 diabetes, ubiquinol supplementation (100–200 mg/day) was shown to lower fasting glucose, HbA1c, and insulin resistance.^27^

Variability in clinical outcomes may arise from variations in formulation, dosage, and patient demographics. The higher bioavailability of ubiquinol demonstrated in this study adds pharmacokinetic evidence to the higher therapeutic effects documented with various formulations or in specific patient categories. Nonetheless, these findings should be evaluated carefully until they are corroborated by pharmacodynamic and clinical outcome data.

In the present study, the two formulations had similar safety profiles. There were no serious adverse events reported. This study is in line with earlier safety studies of CoQ10 supplements, which show that they demonstrated safety, even at high doses.^28^ This study has certain limitations. Initially, the study comprised healthy adult volunteers, who may not accurately represent the target populations for CoQ10 supplementation, including elderly individuals or patients with cardiovascular or neurodegenerative disorders. Second, the study was structured as a single‐dose crossover trial and did not evaluate long‐term exposure, steady‐state kinetics, or potential accumulation. The small sample size (n = 12) and low power in baseline‐corrected data (29.1%–34.1%) constrain subpopulation analysis, despite considerable inter‐individual variability.

However, previous clinical studies have consistently reported considerable inter‐individual variability in CoQ10 absorption, regardless of whether the supplement is administered as ubiquinone or ubiquinol. A crossover study by Vitetta et al in 11 healthy volunteers compared the bioavailability of three CoQ10 formulations (ubiquinone 150 mg, ubiquinol 150 mg, and liposomal ubiquinone) over 6 weeks. The study found significant differences in baseline plasma CoQ10 levels between participants, which persisted after supplementation.^29^ This variability could stem from physiological limitations in CoQ10 absorption.^30^

Overall, the results of this randomized crossover study demonstrate that the body assimilates the ubiquinol formulation more effectively than standard ubiquinone. The results suggest that ubiquinol has better pharmacokinetics; however, the findings’ clinical relevance requires further research in larger, more diverse populations to elucidate pharmacodynamic effects and therapeutic benefits.

## Conclusion

In this pilot study involving 12 subjects, the cocrystal formulation resulted in significantly higher C_max_ and AUC_0–t_ compared to ubiquinone. The AUC_0–t_ was twice as high, the C_max_ was more than twice as high, while AUC_0–∞_ demonstrated 3.4‐fold higher total systemic exposure with no significant adverse effects observed. These results are consistent with earlier studies that showed that ubiquinol is more bioavailable than ubiquinone.^18^ These studies further support a stabilized ubiquinol formulation as a promising approach. The cocrystal formulation product uses cocrystal ubiquinol as the ingredient, which successfully overcomes the recognized challenges with ubiquinol instability while greatly increasing absorption, consistent with previous research on the enhanced bioavailability of cocrystal ubiquinol in rats.^22^

The study's small sample size and single‐dose design in healthy adults limit generalizability, with baseline‐corrected data showing low statistical power (29.1% for C_max_, 34.1% for AUC_0–t_) due to high variability (CV% up to 46.2%). However, uncorrected data exhibited higher power (95.8% for AUC_0–t_), suggesting robust preliminary evidence. The augmented bioavailability observed in this study may enhance treatment outcomes in populations with diminished capacity to convert ubiquinone to ubiquinol, including older adults and individuals with chronic illnesses. These findings strongly support the need to advance this formulation into larger clinical trials, where it can be tested on a broader scale to assess its effectiveness. Additionally, this research contributes valuable insights to the field of CoQ10 formulation by highlighting a promising strategy aimed at significantly improving both absorption and overall efficacy.

## Conflicts of Interest

The authors declare no conflicts of interest.

## Funding

This work was supported by Cocrystal Health Industry Co., Ltd, Zhejiang, China.

## Supporting information



Supporting information

Supporting information

Supporting information

Supporting information

Supporting information

## Data Availability

The raw data supporting the conclusion of this article will be made available by the authors without undue reservation.
