# Supplementary material for: A Randomized, Double‐Blind, Two‐Treatment, Two‐Period, Crossover Study Investigating the Systemic Bioavailability of a Novel Cocrystal Ubiquinol Formulation Compared with a Ubiquinone Formulation in Healthy Adults
Source: Clin Pharmacol Drug Dev. 2026 Mar 6;15(3):e70042. doi: 10.1002/cpdd.70042 (PMC12965043; doi:10.1002/cpdd.70042)
Supplement: Supplementary file 2 — Supporting information [file CPDD-15-0-s001.docx]

**Figure S1:** Ping-pong plots of baseline-corrected pharmacokinetic parameters of total ubiquinone following administration of Test (T) and Reference (R) formulations. **(A)** C_max_ values and **(B)** AUC_t_ values for individual subjects across the two treatment periods. Each line represents a subject’s measurements, with randomization to one of two sequences (RT or TR) indicated. The plots illustrate inter-subject variability and allow visual comparison of C_max_ and AUC_t_ trends between the two formulations.
