# Supplementary figures and images for: A Randomized, Double‐Blind, Two‐Treatment, Two‐Period, Crossover Study Investigating the Systemic Bioavailability of a Novel Cocrystal Ubiquinol Formulation Compared with a Ubiquinone Formulation in Healthy Adults
Source: Clin Pharmacol Drug Dev. 2026 Mar 6;15(3):e70042. doi: 10.1002/cpdd.70042 (PMC12965043; doi:10.1002/cpdd.70042)

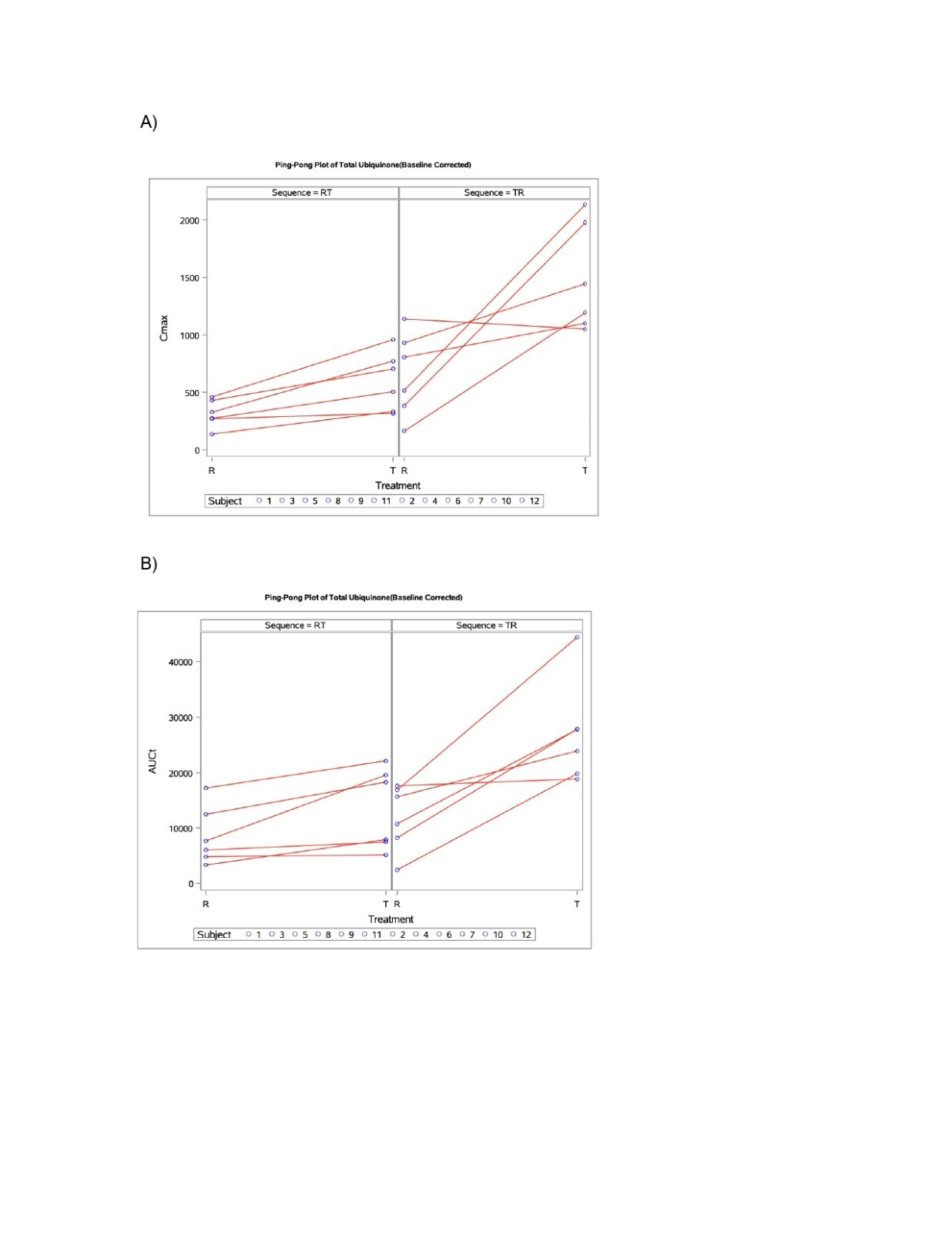

Supplement: Supplementary file 3 — Supporting information [file CPDD-15-0-s002.tiff]
