# Supplementary material for: A Randomized, Double‐Blind, Two‐Treatment, Two‐Period, Crossover Study Investigating the Systemic Bioavailability of a Novel Cocrystal Ubiquinol Formulation Compared with a Ubiquinone Formulation in Healthy Adults
Source: Clin Pharmacol Drug Dev. 2026 Mar 6;15(3):e70042. doi: 10.1002/cpdd.70042 (PMC12965043; doi:10.1002/cpdd.70042)
Supplement: Supplementary file 4 — Supporting information [file CPDD-15-0-s005.docx]

**Supplementary Table S1:** Individual baseline-corrected pharmacokinetic parameters of the test formulation following single-dose administration in healthy subjects.

| **Subject** | **Sequence** | **Period** | **Treatment** | **C_max_ (ng/mL)** | **AUC_t_ (ng·h/mL)** | **AUC_inf_ (ng·h/mL)** | **ln(C_max_)** | **ln(AUC_t_)** | **ln(AUC_inf_)** |
| --- | --- | --- | --- | --- | --- | --- | --- | --- | --- |
| 1 | RT | 2 | T | 506.289 | 7,462.759 | 8,148.749 | 6.227 | 8.918 | 9.006 |
| 2 | TR | 1 | T | 1,101.222 | 23,887.390 | 30,963.662 | 7.004 | 10.081 | 10.341 |
| 3 | RT | 2 | T | 958.310 | 19,553.071 | 34,868.512 | 6.865 | 9.881 | 10.459 |
| 4 | TR | 1 | T | 1,444.962 | 44,402.137 | 106,050.966 | 7.276 | 10.701 | 11.572 |
| 5 | RT | 2 | T | 318.796 | 5,143.429 | 9,293.986 | 5.765 | 8.545 | 9.137 |
| 6 | TR | 1 | T | 1,050.965 | 18,826.436 | 38,773.064 | 6.957 | 9.843 | 10.565 |
| 7 | TR | 1 | T | 1,976.609 | 27,843.464 | 35,693.412 | 7.589 | 10.234 | 10.483 |
| 8 | RT | 2 | T | 333.369 | 7,903.823 | 10,465.381 | 5.809 | 8.975 | 9.256 |
| 9 | RT | 2 | T | 772.512 | 18,276.280 | 30,899.297 | 6.650 | 9.813 | 10.338 |
| 10 | TR | 1 | T | 2,134.306 | 27,769.780 | 45,701.693 | 7.666 | 10.232 | 10.730 |
| 11 | RT | 2 | T | 703.578 | 22,117.960 | 81,202.700 | 6.556 | 10.004 | 11.305 |
| 12 | TR | 1 | T | 1,192.607 | 19,805.432 | 31,575.666 | 7.084 | 9.894 | 10.360 |

Data is presented as individual subject pharmacokinetic parameter values.

Pharmacokinetic parameters were estimated using noncompartmental analysis. Natural log-transformed parameters [ln(C_max)_, ln(AUC_t_), and ln(AUC_inf_)] were used for the statistical assessment of bioavailability.

*Abbreviations:* C_max_, maximum observed plasma concentration; AUC_t_, area under the plasma concentration–time curve from time zero to the last quantifiable concentration; AUC_inf_, area under the plasma concentration–time curve extrapolated to infinity; ln, natural logarithm; T, test formulation; RT/TR, randomized treatment sequence; Period, dosing period in the crossover design.
