# Supplementary material for: A Randomized, Double‐Blind, Two‐Treatment, Two‐Period, Crossover Study Investigating the Systemic Bioavailability of a Novel Cocrystal Ubiquinol Formulation Compared with a Ubiquinone Formulation in Healthy Adults
Source: Clin Pharmacol Drug Dev. 2026 Mar 6;15(3):e70042. doi: 10.1002/cpdd.70042 (PMC12965043; doi:10.1002/cpdd.70042)
Supplement: Supplementary file 5 — Supporting information [file CPDD-15-0-s004.docx]

**Supplementary Table S2:** Individual baseline-uncorrected pharmacokinetic parameters of the test formulation following single-dose administration in healthy subjects.

| **Subject** | **Sequence** | **Period** | **Treatment** | **C_max_ (ng/mL)** | **AUC_t_ (ng·h/mL)** | **AUC_inf_ (ng·h/mL)** | **ln(C_max_)** | **ln(AUC_t_)** | **ln(AUC_inf_)** |
| --- | --- | --- | --- | --- | --- | --- | --- | --- | --- |
| 1 | RT | 2 | T | 1,446.250 | 52,380.692 | 202,185.236 | 7.277 | 10.866 | 12.217 |
| 2 | TR | 1 | T | 1,907.592 | 62,593.150 | 171,866.259 | 7.554 | 11.044 | 12.054 |
| 3 | RT | 2 | T | 1,816.976 | 60,682.281 | 264,171.016 | 7.505 | 11.013 | 12.484 |
| 4 | TR | 1 | T | 2,573.792 | 98,505.362 | 463,968.317 | 7.853 | 11.498 | 13.048 |
| 5 | RT | 2 | T | 1,463.327 | 61,803.060 | 227,042.787 | 7.288 | 11.032 | 12.333 |
| 6 | TR | 1 | T | 1,849.927 | 57,099.602 | 313,701.406 | 7.523 | 10.953 | 12.656 |
| 7 | TR | 1 | T | 2,533.873 | 54,573.065 | 111,103.847 | 7.838 | 10.907 | 11.618 |
| 8 | RT | 2 | T | 767.451 | 28,640.810 | 83,998.618 | 6.643 | 10.263 | 11.339 |
| 9 | RT | 2 | T | 1,373.821 | 47,016.676 | 163,153.242 | 7.225 | 10.758 | 12.002 |
| 10 | TR | 1 | T | 2,719.190 | 56,077.409 | 142,048.425 | 7.908 | 10.934 | 11.864 |
| 11 | RT | 2 | T | 1,368.357 | 53,972.613 | 407,200.378 | 7.221 | 10.896 | 12.917 |
| 12 | TR | 1 | T | 1,591.475 | 38,826.149 | 103,758.175 | 7.372 | 10.567 | 11.550 |

Data is presented as individual subject pharmacokinetic parameter values.

Pharmacokinetic parameters were estimated using noncompartmental analysis. Natural log-transformed parameters [ln(C_max)_, ln(AUC_t_), and ln(AUC_inf_)] were used for the statistical assessment of bioavailability.

*Abbreviations:* C_max_, maximum observed plasma concentration; AUC_t_, area under the plasma concentration–time curve from time zero to the last quantifiable concentration; AUC_inf_, area under the plasma concentration–time curve extrapolated to infinity; ln, natural logarithm; T, test formulation; RT/TR, randomized treatment sequence; Period, dosing period in the crossover design.
